# Supplementary material for: Discrimination and violence against women migrant workers in Thailand during the COVID-19 pandemic: A mixed-methods study
Source: PLoS One. 2024 May 3;19(5):e0300388. doi: 10.1371/journal.pone.0300388 (PMC11068168; doi:10.1371/journal.pone.0300388)
Supplement: S1 File — (PDF) [file pone.0300388.s001.pdf]

Research on experiences of violence among women migrant workers migrating from Lao PDR, Myanmar, and Cambodia to Thailand

SURVEY WITH WOMEN MIGRANT WORKERS

This survey is to be undertaken with women migrant workers (WMW). The questions are designed to gain an understanding of the prevalence of violence experienced by WMW, the types of violence, the relationships between the victim and perpetrator, and any coping strategies or protective factors WMW make use of to keep themselves safe. These questions are also designed to investigate the experiences of WMW who experience intersecting forms of inequality and discrimination and gauge the impact of COVID-19 on WMW.

The surveys will take place in person and will be conducted by a WMW researcher. The survey participants will be recruited using the WMW researchers' connections, relationships, and networks. All surveys will be conducted using the participant's own language. The WMW researcher will read through the participant information sheet and complete the oral consent form with the survey participants. Oral consent will be gained at the beginning of the survey.

Research Questions:

1.What are WMW experiences of: (a) intimate partner violence, (b) non-partner sexual violence, and (c) workplace abuse, violence and harassment, in different stages of the migration cycle?

2. How do experiences of violence differ across demographic groups (based on age, marital status, country of origin, ethnicity, (dis)ability, etc.), and between sectors and work type?

3.What are the impacts of violence on WMW in the context of labour migration? What are the impacts on WMW's health, employment and livelihood, wellbeing?

4.What are the impacts of violence against WMW more broadly (i.e. on workplaces, sectors, communities, etc)?

5.How do WMW respond to/cope with violence and consequences of violence in the context of labour migration?

8. What are the linkages between WMW's experiences of violence and irregular versus regular migration? What are the linkages between WMW experiences of violence and their access to protections, support (both formal and informal), and services?

9.What are the protective factors that may decrease the chance for WMW to experience violence and/or be at risk of being trafficked?

11.How has COVID-19 impacted on WMW and their experience of violence, particularly in the context of labour migration?

Research on experiences of women migrant workers in Thailand

SURVEY WITH WOMEN MIGRANT WORKERS

Introduction: Read through Participant Information sheet and complete consent forms
Hello, my name is ______________________ . I am here on behalf of UN Women and College of Public Health Sciences, Chulalongkorn University. We are conducting a survey in Thailand to learn about women's experiences of migration to Thailand. This survey will take approximately one hour. Are you happy/willing to participate in this survey?

CONSENT
I have read and understood the Information Sheet describing the Women Migrant Worker study or have had the sheet read to me. I understand that at any time I may withdraw from the project, as well as withdraw any information that I have provided.
I note that this project has been reviewed and approved by the Research Ethics Review Committee for Research Involving Human Research Participants, Group I, Chulalongkorn University.

1. Do you consent to participate in the study?	1. YES<br>2. NO → **END SURVEY**
2. I freely and voluntarily agree to participate as a subject in the project. I consent to publication of the results of the project/the information given to me on the understanding that my anonymity is preserved.	

#	Section 1: Participant's Information		Skip
I would like to begin by asking for your personal details, and this information will help you to understand your experience better. All the information you provide will be kept completely confidential and your name will not be recorded so, no one else will know what information you have given me. Can I start the interview?			
3.	How old are you?	[put the number of completed age]	
4.	In which country were you born?	1. Lao PDR<br>2. Cambodia<br>3. Myanmar<br>4. Thailand<br>5. Other___<br>6. do not know / do not remember<br>7. do not want to answer	
5.	Do you currently have a partner (husband, wife, boyfriend, girlfriend or intended partner) in Thailand?	1. Yes. I have a male partner (husband, boyfriend or intended) in Thailand.<br>2. Yes. I have a female partner (wife, girlfriend or intended) in Thailand.<br>**3. No, currently, I do not have a partner in Thailand.**<br>4. **I do not want to answer.**	 <br><br>→ Q7<br><br>→ Q7
6.	Do you and your partner live together in Thailand?	**1. Live**<br>**2. Do not live**<br>**3. I do not know / do not remember**<br>**4. I do not want to answer**	→ Q11<br>→ Q11<br>→ Q11<br>→ Q11
7.	Do you currently have a partner (husband, wife, boyfriend, girlfriend or intended partner) in your home country or in another country?	1. Yes. I have a male partner (husband, boyfriend or intended partner) in my home country.<br>2. Yes. I have a male partner (husband, boyfriend, or intended partner) in another country.<br>**3.** Yes. I have a female partner (wife, girlfriend or intended partner) in my home country.<br>4. Yes. I have a female partner (wife, girlfriend or intended partner) in another country.<br>**5. No. I do not have a current partner in the home country or in any other country.**<br>6. **I do not want to answer.**	 <br><br><br><br>→ Q9<br><br>→ Q9
8.	Did you and your partner live together while you were not in Thailand?	**1. Yes, we did.**<br>**2. No, we did not stay.**<br>**3. I do not know / I do not remember.**<br>**4. I do not want to answer.**	→ Q11<br>→ Q11<br>→ Q11<br>→ Q11
9.	Have you ever been married or lived with a partner?	**1. I have ever been married to a man.**<br>**2. I used to live with a male partner. But I have never been married with him.**<br>**3. I have ever been married to a woman.**<br>**4. I used to live with a female partner. But I have never been married with her.**<br>5. Never married / never lived.<br>6. I do not know / do not remember.<br>7. I do not want to answer.	→ Q11<br>→ Q11<br><br>→ Q11<br>→ Q11
10.	Have you ever been in a relationship with a partner (engagement or dating, etc.) instead of living together?	1. Yes, with a man.<br>2. Yes, with a woman.<br>3. No<br>4. I do not know / I do not remember<br>5. I do not want to answer	

11.	Do you have a child under 18 who is being cared for (whether or not you are blood-related)?	1. Yes, I have.<br>**2. No, I do not have.**<br>3. **I do not want to answer.**	 → Q15<br>→ Q15
12.	How many children under the age of 18 are you caring for?	[put the number]	
13.	Do they live with you or in your home country?	1. Live in my home country<br>2. Live with me in Thailand<br>3. Live in Thailand, but not with me<br>4. I do not know / I do not remember<br>5. I do not want to answer	
14.	Are you the sole parent or a caregiver?	1. Yes<br>2. No<br>3. I do not know / I do not remember<br>4. I do not want to answer	
15.	Which type(s) of residence do you live in Thailand?	*Please select all that applies.*<br>1. Room / House / Apartment for rent<br>2. Inside the workplace provided by the employer<br>3. Outside the workplace provided by the employer<br>4. Hostel<br>5. Own a house or apartment<br>6. Other _________<br>7. I do not want to answer	
16.	Currently in Thailand, with whom do you live with?	*Please select all that applies.*<br>1. Alone<br>2. With a partner<br>3. With my children<br>4. With my relatives<br>5. With my husband's relatives<br>6. With others (not relatives)<br>7. Other ____________<br>*8.* I do not want to answer	
17.	What country did you live in before coming to Thailand?	1. Lao PDR<br>2. Cambodia<br>3. Myanmar<br>4. Other___<br>5. do not know / do not remember<br>6. do not want to answer	
18.	How many times have you come to Thailand to work? For example, returning home or going to another country to work and then returning to Thailand to work.<br>*Note: please consider the times of migrating for working. Do not consider the other reasons, such as for education, or for marriage.*	1. Only once: This is my first time coming to Thailand to work.<br>2. Twice<br>3. Twice or more<br>4. Many times<br>5. I do not know / do not remember<br>6. I do not want to answer	
19.	Overall, how long have you been in Thailand? If you have lived in Thailand more than once, please count those times as well.	1. Under 12 months<br>2. 12 months to 2 years<br>3. 3 to 5 years<br>4. 5 years and above<br>5. I do not know / do not remember<br>6. I do not want to answer	
20.	How did you come to Thailand this time?	1. By MOU<br>2. Through a broker or employment agency<br>3. With Certificate of Accreditation (CI)	

		4. Through unrecognized border gates<br>5. Other: _______<br>6. I do not know / do not remember<br>7. I do not want to answer	
21.	Can you speak in Thai?	1. I can speak fluently.<br>2. I can speak a little bit.<br>3. I cannot speak.<br>4. I do not want to answer	
22.	Can you read Thai?	1. I can speak fluently.<br>2. I can speak a little bit.<br>3. I cannot speak.<br>4. I do not want to answer	
23.	What kind of work do you usually do in Thailand?	* *Please select all appropriate ones.*<br>1. Food trade<br>2. Garment factory work<br>3. Other factory works<br>4. Construction<br>5. Agriculture<br>6. Fisheries<br>7. Seafood production<br>8. Other food production<br>9. Housework / Help<br>10. Entertainment<br>11. Cleaning<br>12. Care taking<br>13. Massage<br>14. Karaoke shop worker<br>15. Sex work<br>16. Housework / Housewife (unpaid)<br>17. Unemployed / Not working / Student<br>18. Other _______<br>19. I do not know / do not remember<br>20. I do not want to answer	
24.	What kind of work did you usually do in your home country?	* Select all appropriate ones<br>1. Food trade<br>2. Garment factory work<br>3. Other factory works<br>4. Construction<br>5. Agriculture<br>6. Fisheries<br>7. Seafood production<br>8. Other food production<br>9. Paid housework / help<br>10. Entertainment<br>11. Cleaning<br>12. Care taking<br>13. Massage<br>14. Karaoke shop worker<br>15. Sex work<br>16. Housework / Housewife (unpaid)<br>17. Professional work - Government workers/ Doctor/ Nurses/ Teacher<br>18. Office worker/ Secretary/ Technology/ Bank staff<br>19. Trading / Self-employment<br>20. Armed Forces: Police; Soldier And so on<br>21. Driver / Rental Driver<br>22. Unemployed / Not working / Student	

		23. Other _______<br>24. I do not know / do not remember<br>25. I do not want to answer	
25.	How often did you earn a month before the Covid-19 pandemic (before March 2020)?	1. Under 500 baht<br>2. 501 to 2,500 baht<br>3. 2,501 to 5,000 baht<br>4. 5,001 to 10,000 baht<br>5. 10,001 to 15,000 baht<br>6. 15,001 to 30,000 baht<br>7. 30,001 baht and above<br>8. I do not know / do not remember<br>9. I do not want to answer	
26.	Have you worked or earned income in the last 12 months?	1. Yes, I have<br>**2. No, I have not done.**<br>3. I do not know / do not remember<br>4. I do not want to answer	→ Q28
27.	How much do you earn per month in your current or most recent job?	1. Under 500 baht<br>2. 501 to 2,500 baht<br>3. 2,501 to 5,000 baht<br>4. 5,001 to 10,000 baht<br>5. 10,001 to 15,000 baht<br>6. 15,001 to 30,000 baht<br>7. 30,001 baht and above<br>8. I do not know / do not remember<br>9. I do not want to answer	
28.	Do you work all year round or seasonally or for long periods of time?	1. All year round<br>2. Seasonally<br>3. Once in a while (not fixed)<br>4. No / does not work often<br>5. I do not know / do not remember<br>6. I do not want to answer	
29.	Do you get paid regularly in your current job or your most recent job?	1. Always get<br>2. Usually<br>3. Sometimes<br>4. Rare<br>5. Never<br>6. I do not know / do not remember<br>7. I do not want to answer	
30.	In your current or most recent job, how do you get paid your salary? (directly to you/ indirectly from someone else)	1. Give it to me directly<br>2. Give directly to others<br>3. Give directly to me, but I have to give to others<br>4. I do not know / do not remember<br>5. I do not want to answer	
31.	What is the highest level of education you have ever received?	1. No education<br>2. I started primary school but did not finish it<br>3. Completed primary school<br>4. I started middle school but did not finish<br>5. Completed middle school<br>6. I went to university but did not graduate<br>7. Graduated with a bachelor's degree<br>8. Postgraduate<br>9. I do not know / do not remember<br>10. I do not want to answer	
32.	Are you currently pregnant? Or have you been pregnant in the last 12 months?	1. Yes. I am currently pregnant<br>2. I am not pregnant at the moment but have had it in the last 12 months<br>3. No, I have not ever been pregnant.	

		4. I do not know / I do not remember<br>5. I do not want to answer	
33.	Do you define yourself as a person with different sexual orientation/ gender identity / gender expression - LGBTQ? (gay/ lesbian/ bisexual, a person of the opposite sex dress/ a person of both sexes/<br>A person who does not define himself as a man or a woman by two gender criteria)	1. Yes, I define myself<br>2. No, I do not define myself<br>3. I do not know / do not remember<br>4. I do not want to answer	
34.	Do you define yourself as a member of an ethnic minority in your home country?	1. Yes, I define myself<br>2. No, I do not define myself<br>3. I do not know / do not remember<br>4. I do not want to answer	
The next set of questions covers health issues; You will be asked about the difficulties you may encounter in performing certain actions due to the situation.			
35.	Do you have vision problems even if you wear glasses?	1. I do not have any difficulties.<br>2. Yes. I have some difficulties.<br>3. Yes. I have many difficulties.<br>4. Yes. I cannot do anything because of difficulties.<br>5. I do not know / do not remember<br>6. I do not want to answer.	
36.	Do you have hearing problems even if you wear hearing aids?	1. I do not have any difficulties.<br>2. Yes. I have some difficulties.<br>3. Yes. I have many difficulties.<br>4. Yes. I cannot do anything because of difficulties.<br>5. I do not know / do not remember<br>6. I do not want to answer.	
37.	Do you have any difficulties in walking or climbing stairs?	1. I do not have any difficulties.<br>2. Yes. I have some difficulties.<br>3. Yes. I have many difficulties.<br>4. Yes. I cannot do anything because of difficulties.<br>5. I do not know / do not remember<br>6. I do not want to answer.	
38.	Do you have any difficulties in remembering or concentrating for long periods of time?	1. I do not have any difficulties.<br>2. Yes. I have some difficulties.<br>3. Yes. I have many difficulties.<br>4. Yes. I cannot do anything because of difficulties.<br>5. I do not know / do not remember<br>6. I do not want to answer.	
39.	Do you have any difficulties in taking care of yourself? (Cleaning or dressing)	1. I do not have any difficulties.<br>2. Yes. I have some difficulties.<br>3. Yes. I have many difficulties.<br>4. Yes. I cannot do anything because of difficulties.<br>5. I do not know / do not remember<br>6. I do not want to answer.	
40.	Do you have any difficulties in communicating in your (mother) language? For example, you do not understand what the other person is	1. I do not have any difficulties.<br>2. Yes. I have some difficulties.<br>3. Yes. I have many difficulties.<br>4. Yes. I cannot do anything because of difficulties.	

	saying or that the other person does not understand what you are saying.	5. I do not know / do not remember<br>6. I do not want to answer.	

#	Section 2: Gender Attitudes		Skip
People have different attitudes on men and women; families; and acceptable behaviors for men and women at home I will read you some sentences. Please tell us how much you agree or disagree with this statement. The choices are strongly agree; agree; disagree; and strongly disagree. There is no right or wrong for the answer.			
41.	A married woman must obey the instructions of her husband.	1. Strongly agree<br>2. Agree<br>3. Disagree<br>4. Strongly disagree<br>5. I do not know / do not remember<br>6. I do not want to answer	
42.	A married woman cannot refuse to have sex with her husband.	1. Strongly agree<br>2. Agree<br>3. Disagree<br>4. Strongly disagree<br>5. I do not know / do not remember<br>6. I do not want to answer	
43.	If a married woman earns more than her husband, it is bad for their relationship.	1. Strongly agree<br>2. Agree<br>3. Disagree<br>4. Strongly disagree<br>5. I do not know / do not remember<br>6. I do not want to answer	
Thank you. I would like to ask a few more questions about your opinions on men and women.			
44.	If a husband abuses his wife, the wife is to be blamed.	1. Strongly agree<br>2. Agree<br>3. Disagree<br>4. Strongly disagree<br>5. I do not know / do not remember<br>6. I do not want to answer	
45.	A woman has to endure violence from her partner to take care of her family.	1. Strongly agree<br>2. Agree<br>3. Disagree<br>4. Strongly disagree<br>5. I do not know / do not remember<br>6. I do not want to answer	
46.	If a wife is beaten by her husband, she can tell others.	1. Strongly agree<br>2. Agree<br>3. Disagree<br>4. Strongly disagree<br>5. I do not know / do not remember<br>6. I do not want to answer	
47.	Women who have been sexually abused should address the issues themselves rather than complaining to officials.	1. Strongly agree<br>2. Agree<br>3. Disagree<br>4. Strongly disagree<br>5. I do not know / do not remember<br>6. I do not want to answer	
48.	If a woman is raped, it is usually her fault that has caused such a situation.	1. Strongly agree<br>2. Agree<br>3. Disagree<br>4. Strongly disagree<br>5. I do not know / do not remember<br>6. I do not want to answer	

#	Section 3: Impact of COVID-19 pandemic		Skip
	In this section, I would like to ask you about the COVID-19 epidemic and how it has affected your life. The following are some of the questions you may have encountered during the period from March 2020 to the present.		
49.	Where did you live in late March 2020?	1. Bangkok<br>2. Chiang Mai<br>3. Elsewhere in Thailand<br>4. Lao People's Democratic Republic<br>5. Cambodia<br>6. Myanmar<br>7. Other ______<br>8. I do not know / do not remember<br>9. I do not want to answer	
50.	Have you been traveling or trying to travel somewhere since March 2020? Could you tell me the destination?	**1. I do not go**<br>2. Country of residence<br>3. Thailand<br>4. Elsewhere in Thailand<br>5. Other than Thailand and country of origin<br>6. I do not know / do not remember<br>7. I do not want to answer	→Q52
51.	What were the reasons for your efforts to travel?	** Select all appropriate ones*<br>1. Find a job<br>2. Feeling insecure<br>3. Because I want to be with my family / friends<br>4. I want to go home<br>5. I lost my job<br>6. Due to family pressure<br>7. Visa / Certificate expires<br>8. Border blockade<br>9. Discrimination in Thailand<br>10. Discrimination in the motherland<br>11. Other _____<br>12. I do not know / do not remember<br>13. I do not want to answer	
52.	Did anyone treat you badly or discriminate against you during the Coronavirus Outbreak (March 2020 until now)?	1. Yes<br>**2. No**<br>**3. I do not know / do not remember**<br>**4. I do not want to answer**	 →Q58<br>→Q58<br>→Q58
53.	Why do you think they treated you badly or discriminated against you?<br><br>(A) Do you think that they discriminated against or treated badly you because you were a migrant worker?<br><br>(B) Do you think they have treated you badly or discriminated against you because of your age?<br><br>(C) Do you think that because you are a woman, they have treated you badly or discriminated against you?	1. I think so 2. I do not think so<br>3. I do not know / do not remember<br>4. I do not want to answer<br><br>1 2 3 4<br><br>1 2 3 4<br><br>1 2 3 4	

Assessment: If you do not have any disabilities, skip to Question Q55.			
54.	Do you think they treat you badly or discriminate against you because you have a disability?	1. I think so<br>2. I do not think so<br>3. I do not know / do not remember<br>4. I do not want to answer	
Assessment: If you do not define yourself as a person with different sexual orientation/ gender identity / gender expression – LGBTQ (gay/ lesbian/ bisexual, a person of the opposite sex dress/ a person of both sexes/ A person who does not define himself as a man or a woman by two gender criteria), please skip to Question Q56.			
55.	Do you think that you are being discriminated against because of your sexual orientation and / or sexual orientation?	1. I think so<br>2. I do not think so<br>3. I do not know / do not remember<br>4. I do not want to answer	
Assessment: If you are not an ethnic minority, please skip to Question Q57.			
56.	Do you think that you are being discriminated against or being treated badly because you belong to an ethnic minority?	1. I think so<br>2. I do not think so<br>3. I do not know / do not remember<br>4. I do not want to answer	
57.	Where did you experience such abusive treatment or discrimination? In Thailand? In your home country? Or while traveling?	*Choose all the appropriate answers*<br>1. Thailand<br>2. Home country<br>3. While traveling<br>4. I do not know / do not remember<br>5. I do not want to answer	
58.	Did you lost your job because of COVID-19 pandemic?	1. Lost<br>2. Not lost<br>3. I do not know / do not remember<br>4. I do not want to answer	
59.	Did you lose your income during the COVID-19 pandemic compared to your usual income?	1. Lost<br>2. Not lost<br>3. I do not know / do not remember<br>4. I do not want to answer	
Assessment: If you did not lose your job or income during the epidemic, please skip to Question Q63.			
60.	Does losing a job or income mean that you are dependent on your partner or others for financial support?	1. Yes<br>2. No.<br>3. I do not know / do not remember<br>4. I do not want to answer	
61.	Does losing a job or income mean that you have to take out a loan or get into debt?	1. Yes<br>2. No.<br>3. I do not know / do not remember<br>4. I do not want to answer	
62.	Does losing a job or income mean that you cannot support those who depend on you?	1. Yes<br>2. No.<br>3. I do not know / do not remember<br>4. I do not want to answer	
63.	Have you ever been in a country in which travelling was restricted (shops and businesses were closed to prevent the spread of COVID-19, and many people had to live in their homes - Lockdown)?	1. Yes<br>**2. No.**<br>**3. I do not know / do not remember**<br>**4. I do not want to answer**	 →Q65<br>→Q65<br>→Q65
64.	If so, in what country?	1. [Enter country name]<br>2. I do not know / do not remember<br>3. I do not want to answer	
65.	Have you ever been subject to government-mandated quarantine in a	1. Yes<br>**2. No.**<br>**3. I do not know / do not remember**	 →Q67<br>→Q67

	country (eg. hostels, hotels, or government facility quarantine)?	**4. I do not want to answer**		→Q67
66.	If so, in what country?	1. [Enter country name]<br>2. I do not know / do not remember<br>3. I do not want to answer		
Skip to question no. 67: If you have never been in lockdown or in quarantine (if Q63=2 or 3 or 4 , and Q65=2 or 3 or 4, please skip to question S4)				
67.	Have you ever had any side effects from living in lockdown or in quarantine?	A. I had to take a break from work	1. Yes<br>2. No<br>3. I do not know / do not remember<br>4. I do not want to answer	
		B. Lose a job / Changing an employer / job	1. Yes<br>2. No<br>3. I do not know / do not remember<br>4. I do not want to answer	
		C. Financial stress (struggling to pay rent, food, medical bills, etc.)	1. Yes<br>2. No<br>3. I do not know / do not remember<br>4. I do not want to answer	
		D. Migration status changes or documents expire	1. Yes<br>2. No<br>3. I do not know / do not remember<br>4. I do not want to answer	
		E. be deported	1. Yes<br>2. No<br>3. I do not know / do not remember<br>4. I do not want to answer	
		F. feeling ashamed/ humiliated	1. Yes<br>2. No<br>3. I do not know / do not remember<br>4. I do not want to answer	
		G. feeling scared/ insecure	1. Yes<br>2. No<br>3. I do not know / do not remember<br>4. I do not want to answer	
		H. Children are cared for by someone else	1. Yes<br>2. No<br>3. I do not know / do not remember<br>4. I do not want to answer	
		I. Concerned about children	1. Yes<br>2. No<br>3. I do not know / do not remember<br>4. I do not want to answer	
		J. Physical injury or illness	1. Yes<br>2. No<br>3. I do not know / do not remember<br>4. I do not want to answer	

		K. Lack of access to medical care	1. Yes<br>2. No<br>3. I do not know / do not remember<br>4. I do not want to answer	
		L. Others	[Enter text]	
68.	Did you feel safe in Lockdown or Quarantine? Always? Most of the time? Sometime? Is it rare? Never felt that way?	1. Always<br>2. Most of the time<br>3. Sometimes<br>4. Rarely feel safe<br>5. Never feel<br>6. I do not know / do not remember<br>7. I do not want to answer		
Assessment: If you felt safe, (Q68=1 or 2), please skip to Q70.				
69.	What made you feel insecure?	** Select all appropriate ones*<br>1. Being with a male companion<br>2. Being with male relatives<br>3. Being with a female companion<br>4. Being with female relatives<br>5. Being with friends<br>6. Being with non-relatives<br>7. Because of the living space I live in<br>8. Uncertainty or not understanding about the virus<br>9. Stress or anxiety for the future<br>10. Concerns about finances<br>11. Concerns about being threatened or physically harmed<br>12. Concerns about my visa status<br>13. Concerns about being deported<br>14. Other __________<br>15. I do not know / do not remember<br>16. I do not want to answer		
Assessment: If you felt unsafe (if question Q68= 4 or 5), please skip to S4.				
70.	What made you feel safe?	** Select all appropriate ones*<br>1. Being with or supported by a male companion<br>2. Staying close to or assisted by male relatives<br>3. Being with or supported by a female partner<br>4. Being with or supported by female relatives<br>5. Being with or supported by friends<br>6. Being with or supported by non-relatives<br>7. Being with or assisted by other female migrant workers<br>8. Sharing information through social media or social networks<br>9. Support from non-governmental organizations or migrant groups<br>10. Support from the union<br>11. Where I live<br>12. Other __________<br>13. I do not know / do not remember<br>14. I do not want to answer		

#	Section 4: General Safety		Skip
In this section, I would like to ask you about your experiences and feelings over different periods of time. These questions were asked before the COVID-19 pandemic. Applicable for any period during or after this period.			
71.	How safe did you feel on your migration/travelling to Thailand?	1. Very safe<br>2. Safe<br>3. It is secure nor insecure<br>4. It is not safe<br>5. Very insecure<br>6. I do not know / do not remember<br>7. I do not want to answer	
72.	How safe do you feel in your workplace?	1. Very safe<br>2. Safe<br>3. It is secure nor insecure<br>4. It is not safe<br>5. Very insecure<br>6. I do not know / do not remember<br>7. I do not want to answer	
73.	How safe do you feel in your current residence in Thailand?	1. Very safe<br>2. Safe<br>3. It is secure nor insecure<br>4. It is not safe<br>5. Very insecure<br>6. I do not know / do not remember<br>7. I do not want to answer	
74.	How safe do you feel to commute to / from your current / most recent job?	1. Very safe<br>2. Safe<br>3. It is secure nor insecure<br>4. It is not safe<br>5. Very insecure<br>6. I do not know / do not remember<br>7. I do not want to answer	
**Assessment: If you are not with a partner, pleaseskip to the introduction of question Q125**			
People often share their good or bad feelings if they get married/ live together/ have a loving relationship together. Now I would like to ask you some questions about your current or past relationships and how your husband / partner / boyfriend / girlfriend treated you. If someone interrupts us while I am asking, I will change the subject. I would like to reaffirm that your answers are kept confidential and that you do not have to answer any questions you do not wish to answer. Can I still ask?			
75.	Have you ever been restricted by your (any) partner from working, going to work, trading, making money, or doing plans to get income?	1. Yes<br>**2. No**<br>**3. I do not know / do not remember**<br>4. **I do not want to answer**	 →Q78<br>→Q78<br>→Q78
76.	How many times have those events happened? (once/ a few times/ many times)	1. Once<br>2. A few times<br>3. Many times<br>4. I do not know / do not remember<br>5. I do not want to answer	
77.	Did this happen in the last 12 months (March 2020 until now) or before the past 12 months or both?	1. Only in the last 12 months (March 2020 until now)<br>2. Only before 12 months ago<br>3. Both in the last 12 months and within the last 12 months<br>4. I do not know / do not remember<br>5. I do not want to answer	
78.	Have you ever had your partner forcibly take your income against your will? Or have that person ever had your income taken away from you?	1. There has been<br>**2. Never**<br>**3. I do not know / do not remember**<br>**4. I do not want to answer**	 →Q81<br>→Q81<br>→Q81

79.	How many times have those events happened? (once/ a few times/ many times)	1. Once<br>2. A few times<br>3. Many times<br>4. I do not know / do not remember<br>5. I do not want to answer	
80.	Did this happen in the last 12 months (March 2020 until now) or before the past 12 months or both?	1. Only in the last 12 months (March 2020 until now)<br>2. Only before 12 months ago<br>3. Both in the last 12 months and within the last 12 months<br>4. I do not know / do not remember<br>5. I do not want to answer	
81.	Have you ever been kicked out of your home / living space by one of your partners?	1. Yes<br>**2. No**<br>**3. I do not know / do not remember**<br>4. **I do not want to answer**	 →Q84<br>→Q84<br>→Q84
82.	Would you say that such kind of event happened one time or a few times or many times?	1. Once<br>2. A few times<br>3. Many times<br>4. I do not know / do not remember<br>5. I do not want to answer	
83.	Did this happen in the last 12 months (March 2020 until now) or before the past 12 months or both?	1. Only in the last 12 months (March 2020 until now)<br>2. Only before 12 months ago<br>3. Both in the last 12 months and within the last 12 months<br>4. I do not know / do not remember<br>5. I do not want to answer	
Assessment: If you have never experienced economic violence, skip to Q85			
84.	Who commit such kinds of violence you have said? (Question related to Q75, Q78 and Q81).	1. Current partner<br>2. Former partner<br>3. Both current and former partners<br>4. I do not know / do not remember<br>5. I do not want to answer	
85.	How much control do you have over spending the money you have earned?	1. I have full control<br>2. I usually have control<br>3. I have some control, but often someone else decides<br>4. I have no control; Someone else always decides<br>5. I do not know / do not remember<br>6. I do not want to answer	
The next questions will be about what happens to many women and what your current or former partner may have done to you.			
86.	Have you ever been insulted or harassed by one of your partners by sending you a text message, with social media messages or phone calls.	1. Yes<br>**2. No**<br>**3. I do not know / do not remember**<br>**4. I do not want to answer**	 →Q89<br>→Q89<br>→Q89
87.	Would you say that such kind of event happened one time or a few times or many times?	1. Once<br>2. A few times<br>3. Many times<br>4. I do not know / do not remember<br>5. I do not want to answer	
88.	Did this happen in the last 12 months (March 2020 until now) or before the past 12 months or both?	1. Only in the last 12 months (March 2020 until now)<br>2. Only before 12 months ago	

		3. Both in the last 12 months and within the last 12 months<br>4. I do not know / do not remember<br>5. I do not want to answer	
89.	Have you ever been threatened by any of your partners via text message, email or social media?	1. Yes<br>**2. No**<br>**3. I do not know / do not remember**<br>**4. I do not want to answer**	 →Q92<br>→Q92<br>→Q92
90.	Would you say that such kind of event happened one time or a few times or many times?	1. Once<br>2. A few times<br>3. Many times<br>4. I do not know / do not remember<br>5. I do not want to answer	
91.	Did this happen in the last 12 months (March 2020 until now) or before the past 12 months or both?	1. Only in the last 12 months (March 2020 until now)<br>2. Only before 12 months ago<br>3. Both in the last 12 months and within the last 12 months<br>4. I do not know / do not remember<br>5. I do not want to answer	
Assessment: if you have never encountered harassment or cyber bullying, please skip to Q93.			
92.	Who commit such kinds of violence you have said? (Question related to Q86, and Q89).	1. Current partner<br>2. Former partner<br>3. Both current and former partners<br>4. I do not know / do not remember<br>5. I do not want to answer	
93.	Have you ever been experienced any of the followings done by any of your partners: Insulting you; Making yourself feel bad; Saying something to embarrass you in front of others?	1. Yes<br>**2. No**<br>**3. I do not know / do not remember**<br>**4. I do not want to answer**	 →Q96<br>→Q96<br>→Q96
94.	Would you say that such kind of event happened one time or a few times or many times?	1. Once<br>2. A few times<br>3. Many times<br>4. I do not know / do not remember<br>5. I do not want to answer	
95.	Did this happen in the last 12 months (March 2020 until now) or before the past 12 months or both?	1. Only in the last 12 months (March 2020 until now)<br>2. Only before 12 months ago<br>3. Both in the last 12 months and within the last 12 months<br>4. I do not know / do not remember<br>5. I do not want to answer	
96.	Have you ever been experienced any of the followings done by any of your partners: Destroy things that are important to you; Scaring you; Making you feel like you are being manipulated?	1. Yes<br>**2. No**<br>**3. I do not know / do not remember**<br>**4. I do not want to answer**	 →Q99<br>→Q99<br>→Q99
97.	Would you say that such kind of event happened one time or a few times or many times?	1. Once<br>2. A few times<br>3. Many times<br>4. I do not know / do not remember<br>5. I do not want to answer	
98.	Did this happen in the last 12 months (March 2020 until now) or before the past 12 months or both?	1. Only in the last 12 months (March 2020 until now)<br>2. Only before 12 months ago	

		3. Both in the last 12 months and within the last 12 months<br>4. I do not know / do not remember<br>5. I do not want to answer	
99.	Have you ever had a partner threaten to harm you or someone you care about?	1. Yes<br>**2. No**<br>**3. I do not know / do not remember**<br>**4. I do not want to answer**	 →Q102<br>→Q102<br>→Q102
100.	Would you say that such kind of event happened one time or a few times or many times?	1. Once<br>2. A few times<br>3. Many times<br>4. I do not know / do not remember<br>5. I do not want to answer	
101.	Did this happen in the last 12 months (March 2020 until now) or before the past 12 months or both?	1. Only in the last 12 months (March 2020 until now)<br>2. Only before 12 months ago<br>3. Both in the last 12 months and within the last 12 months<br>4. I do not know / do not remember<br>5. I do not want to answer	
Assessment: If you have never experienced any of psychological violence, please skip to Question Q103			
102.	Who commit such kinds of violence you have said? (Question related to Q93, Q96, and Q99).	1. Current partner<br>2. Former partner<br>3. Both current and former partners<br>4. I do not know / do not remember<br>5. I do not want to answer	
103.	Have you ever been experienced any of the followings done by any of your partners: Slapping you on the cheek; Throwing something that could injure you; Pushing you; Locking you up; Forcefully pulling your hairs?	1. Yes<br>**2. No**<br>**3. I do not know / do not remember**<br>**4. I do not want to answer**	 →Q106<br>→Q106<br>→Q106
104.	Would you say that such kind of event happened one time or a few times or many times?	1. Once<br>2. A few times<br>3. Many times<br>4. I do not know / do not remember<br>5. I do not want to answer	
105.	Did this happen in the last 12 months (March 2020 until now) or before the past 12 months or both?	1. Only in the last 12 months (March 2020 until now)<br>2. Only before 12 months ago<br>3. Both in the last 12 months and within the last 12 months<br>4. I do not know / do not remember<br>5. I do not want to answer	
106.	Have you ever been experienced any of the followings done by any of your partners: Punching you; Beating you with something that hurts you; Dragging you; Beating you?	1. Yes<br>**2. No**<br>**3. I do not know / do not remember**<br>**4. I do not want to answer**	 →Q109<br>→Q109<br>→Q109
107.	Would you say that such kind of event happened one time or a few times or many times?	1. Once<br>2. A few times<br>3. Many times<br>4. I do not know / do not remember<br>5. I do not want to answer	
108.	Did this happen in the last 12 months (March 2020 until now) or before the past 12 months or both?	1. Only in the last 12 months (March 2020 until now)<br>2. Only before 12 months ago	

		3. Both in the last 12 months and within the last 12 months<br>4. I do not know / do not remember<br>5. I do not want to answer	
109.	Have you ever been experienced any of the followings done by any of your partners: Strangulation; Intentionally burning you?	1. Yes<br>**2. No**<br>**3. I do not know / do not remember**<br>**4. I do not want to answer**	 →Q112<br>→Q112<br>→Q112
110.	Would you say that such kind of event happened one time or a few times or many times?	1. Once<br>2. A few times<br>3. Many times<br>4. I do not know / do not remember<br>5. I do not want to answer	
111.	Did this happen in the last 12 months (March 2020 until now) or before the past 12 months or both?	1. Only in the last 12 months (March 2020 until now)<br>2. Only before 12 months ago<br>3. Both in the last 12 months and within the last 12 months<br>4. I do not know / do not remember<br>5. I do not want to answer	
112.	Have you ever been experienced any of the followings done by any of your partners: Using or threatening with a knife or other weapon?	1. Yes<br>**2. No**<br>**3. I do not know / do not remember**<br>**4. I do not want to answer**	 →Q115<br>→Q115<br>→Q115
113.	Would you say that such kind of event happened one time or a few times or many times?	1. Once<br>2. A few times<br>3. Many times<br>4. I do not know / do not remember<br>5. I do not want to answer	
114.	Did this happen in the last 12 months (March 2020 until now) or before the past 12 months or both?	1. Only in the last 12 months (March 2020 until now)<br>2. Only before 12 months ago<br>3. Both in the last 12 months and within the last 12 months<br>4. I do not know / do not remember<br>5. I do not want to answer	
Assessment: If you have never experienced physical violence, please skip to Q116.			
115.	Who commit such kinds of violence you have said? (Question related to Q103, Q106, Q109, and Q99).	1. Current partner<br>2. Former partner<br>3. Both current and former partners<br>4. I do not know / do not remember<br>5. I do not want to answer	
116.	A partner (husband, boyfriend, intended partner, wife, or girlfriend) may tell you to have sex when you do not want to. For example, by threatening or coercing you; Have you ever been forced to do so?<br>*If necessary, please mention this fact: Sexual intercourse is defined as vaginal, oral or anal penetration.*	1. Yes<br>**2. No**<br>**3. I do not know / do not remember**<br>**4. I do not want to answer**	 →Q119<br>→Q119<br>→Q119
117.	Would you say that such kind of event happened one time or a few times or many times?	1. Once<br>2. A few times<br>3. Many times<br>4. I do not know / do not remember<br>5. I do not want to answer	

118.	Did this happen in the last 12 months (March 2020 until now) or before the past 12 months or both?	1. Only in the last 12 months (March 2020 until now)<br>2. Only before 12 months ago<br>3. Both in the last 12 months and within the last 12 months<br>4. I do not know / do not remember<br>5. I do not want to answer	
119.	Have you ever had unintentional sexual intercourse because you were afraid of what your partner might do to you if you refused?	1. Yes<br>**2. No**<br>**3. I do not know / do not remember**<br>**4. I do not want to answer**	 →Q122<br>→Q122<br>→Q122
120.	Would you say that such kind of event happened one time or a few times or many times?	1. Once<br>2. A few times<br>3. Many times<br>4. I do not know / do not remember<br>5. I do not want to answer	
121.	Did this happen in the last 12 months (March 2020 until now) or before the past 12 months or both?	1. Only in the last 12 months (March 2020 until now)<br>2. Only before 12 months ago<br>3. Both in the last 12 months and within the last 12 months<br>4. I do not know / do not remember<br>5. I do not want to answer	
122.	Have you ever been forced by your partner to do any sexual acts: that you do not want to; that affects your dignity; that is disgusting; or that is shameful?	1. Yes<br>**2. No**<br>**3. I do not know / do not remember**<br>**4. I do not want to answer**	 →Q125<br>→Q125<br>→Q125
123.	Would you say that such kind of event happened one time or a few times or many times?	1. Once<br>2. A few times<br>3. Many times<br>4. I do not know / do not remember<br>5. I do not want to answer	
124.	Did this happen in the last 12 months (March 2020 until now) or before the past 12 months or both?	1. Only in the last 12 months (March 2020 until now)<br>2. Only before 12 months ago<br>3. Both in the last 12 months and within the last 12 months<br>4. I do not know / do not remember<br>5. I do not want to answer	
We understand that some of these questions may be difficult for you to answer. Thank you very much for your answers. Your answers really matter.			
Assessment: If you have never experienced any of the above-mentioned violence, please skip to the introduction of Q126.			
125.	Is your husband / partner's relationship with you one of the reasons you decided to move to Thailand? Is it absolutely not, or somewhat yes, or absolutely yes?	1. Absolutely not<br>2. Somewhat yes<br>3. Absolutely yes<br>4. Not applicable / No partner<br>5. I do not know / do not remember<br>6. I do not want to answer	
Now I would like to ask you about other experiences that you did not want to experience. Please think about any men and women. (For women who have had a partner before – please mention "exclude your partner"). Your answers will be kept confidential. you may not be able to answer any questions you do not wish to answer.			
126.	Have you ever been prohibited by someone from doing any of the followings: seeking job; going to work;	1. Yes<br>**2. No**<br>**3. I do not know / do not remember**	 →Q131<br>→Q131

	trading; earning money; or participating in projects from which you could get income?<br>For women who have ever had a partner, please mention this fact if required: Your partner is not included.	**4. I do not want to answer**	→Q131
127.	Would you say that such kind of event happened one time or a few times or many times?	1. Once<br>2. A few times<br>3. Many times<br>4. I do not know / do not remember<br>5. I do not want to answer	
128.	Who did it?	*Select all that apply*<br>1. A male member of the respondent's family<br>2. Female member of the respondent's family<br>3. Male employer<br>4. Female employer<br>5. Male colleague<br>6. Female colleague<br>7. Male stranger<br>8. Stranger woman<br>9. Migration / immigration Officer<br>10. Jobseeker or broker<br>11. Police officer<br>12. Soldier<br>13. Soldier who is not from the State Army<br>14. Other ___<br>15. I do not know / do not remember<br>16. I do not want to answer	
129.	Did this happen in the last 12 months (March 2020 until now) or before the past 12 months or both?	1. Only in the last 12 months (March 2020 until now)<br>2. Only before 12 months ago<br>3. Both in the last 12 months and within the last 12 months<br>4. I do not know / do not remember<br>5. I do not want to answer	
130.	Where did it happen?	*Select all that apply<br>1. In my home country<br>2. While traveling to your home country and Thailand<br>3. At my workplace in Thailand<br>4. At my place of residence in Thailand<br>5. In public places in Thailand<br>6. On the transport vehicle<br>7. I do not know / do not remember<br>8. I do not want to answer	
131.	Has anyone ever <u>taken your money away</u> from you without your consent? Or have you ever had your income retained?<br>For women who have ever had a partner, please mention this fact if required: Your partner is not included.	1. Yes<br>**2. No**<br>**3. I do not know / do not remember**<br>**4. I do not want to answer**	 →Q136<br>→Q136<br>→Q136
132.	Would you say that such kind of event happened one time or a few times or many times?	1. Once<br>2. A few times<br>3. Many times<br>4. I do not know / do not remember<br>5. I do not want to answer	

133.	Who did it?	*Select all that apply*<br>1. A male member of the respondent's family<br>2. Female member of the respondent's family<br>3. Male employer<br>4. Female employer<br>5. Male colleague<br>6. Female colleague<br>7. Male stranger<br>8. Stranger woman<br>9. Migration / immigration Officer<br>10. Jobseeker or broker<br>11. Police officer<br>12. Soldier<br>13. Soldier who is not from the State Army<br>14. Other ___<br>15. I do not know / do not remember<br>16. I do not want to answer	
134.	Did this happen in the last 12 months (March 2020 until now) or before the past 12 months or both?	1. Only in the last 12 months (March 2020 until now)<br>2. Only before 12 months ago<br>3. Both in the last 12 months and within the last 12 months<br>4. I do not know / do not remember<br>5. I do not want to answer	
135.	Where did it happen?	*Select all that apply.*<br>1. In my home country<br>2. While traveling to your home country and Thailand<br>3. At my workplace in Thailand<br>4. At my place of residence in Thailand<br>5. In public places in Thailand<br>6. On the transport vehicle<br>7. I do not know / do not remember<br>8. I do not want to answer	
136.	Has anyone ever evicted you from your home / place of residence?<br>For women who have ever had a partner, please mention this fact if required: Your partner is not included.	1. Yes<br>**2. No**<br>**3. I do not know / do not remember**<br>**4. I do not want to answer**	 →Q141<br>→Q141<br>→Q141
137.	Would you say that such kind of event happened one time or a few times or many times?	1. Once<br>2. A few times<br>3. Many times<br>4. I do not know / do not remember<br>5. I do not want to answer	
138.	Who did it?	*Select all that apply*<br>1. A male member of the respondent's family<br>2. Female member of the respondent's family<br>3. Male employer<br>4. Female employer<br>5. Male colleague<br>6. Female colleague<br>7. Male stranger<br>8. Stranger woman<br>9. Migration / immigration Officer<br>10. Jobseeker or broker<br>11. Police officer<br>12. Soldier<br>13. Soldier who is not from the State Army	

		14. Other ___<br>15. I do not know / do not remember<br>16. I do not want to answer	
139.	Did this happen in the last 12 months (March 2020 until now) or before the past 12 months or both?	1. Only in the last 12 months (March 2020 until now)<br>2. Only before 12 months ago<br>3. Both in the last 12 months and within the last 12 months<br>4. I do not know / do not remember<br>5. I do not want to answer	
140.	Where did it happen?	*Select all that apply*<br>1. In my home country<br>2. While traveling to your home country and Thailand<br>3. At my workplace in Thailand<br>4. At my place of residence in Thailand<br>5. In public places in Thailand<br>6. On the transport vehicle<br>7. I do not know / do not remember<br>8. I do not want to answer	
141.	Have you ever been experienced any of the followings done by anyone: <u>Insulting you; Making yourself feel bad and depressed; Saying something to embarrass you in front of others</u>?<br>For women who have ever had a partner, please mention this fact if required: Your partner is not included.	1. Yes<br>**2. No**<br>**3. I do not know / do not remember**<br>**4. I do not want to answer**	 →Q146<br>→Q146<br>→Q146
142.	Would you say that such kind of event happened one time or a few times or many times?	1. Once<br>2. A few times<br>3. Many times<br>4. I do not know / do not remember<br>5. I do not want to answer	
143.	Who did it?	*Select all that apply*<br>1. A male member of the respondent's family<br>2. Female member of the respondent's family<br>3. Male employer<br>4. Female employer<br>5. Male colleague<br>6. Female colleague<br>7. Male stranger<br>8. Stranger woman<br>9. Migration / immigration Officer<br>10. Jobseeker or broker<br>11. Police officer<br>12. Soldier<br>13. Soldier who is not from the State Army<br>14. Other ___<br>15. I do not know / do not remember<br>16. I do not want to answer	
144.	Did this happen in the last 12 months (March 2020 until now) or before the past 12 months or both?	1. Only in the last 12 months (March 2020 until now)<br>2. Only before 12 months ago<br>3. Both in the last 12 months and within the last 12 months<br>4. I do not know / do not remember<br>5. I do not want to answer	

145.	Where did it happen?	*Select all that apply*<br>1. In my home country<br>2. While traveling to your home country and Thailand<br>3. At my workplace in Thailand<br>4. At my place of residence in Thailand<br>5. In public places in Thailand<br>6. On the transport vehicle<br>7. I do not know / do not remember<br>8. I do not want to answer	
146.	Has anyone ever <u>confiscated your passport or other important documents</u>?<br>For women who have ever had a partner, please mention this fact if required: Your partner is not included.	1. Yes<br>**2. No**<br>**3. I do not know / do not remember**<br>**4. I do not want to answer**	 →Q151<br>→Q151<br>→Q151
147.	Would you say that such kind of event happened one time or a few times or many times?	1. Once<br>2. A few times<br>3. Many times<br>4. I do not know / do not remember<br>5. I do not want to answer	
148.	Who did it?	*Select all that apply*<br>1. A male member of the respondent's family<br>2. Female member of the respondent's family<br>3. Male employer<br>4. Female employer<br>5. Male colleague<br>6. Female colleague<br>7. Male stranger<br>8. Stranger woman<br>9. Migration / immigration Officer<br>10. Jobseeker or broker<br>11. Police officer<br>12. Soldier<br>13. Soldier who is not from the State Army<br>14. Other ___<br>15. I do not know / do not remember<br>16. I do not want to answer	
149.	Did this happen in the last 12 months (March 2020 until now) or before the past 12 months or both?	1. Only in the last 12 months (March 2020 until now)<br>2. Only before 12 months ago<br>3. Both in the last 12 months and within the last 12 months<br>4. I do not know / do not remember<br>5. I do not want to answer	
150.	Where did it happen?	*Select all that apply*<br>1. In my home country<br>2. While traveling to your home country and Thailand<br>3. At my workplace in Thailand<br>4. At my place of residence in Thailand<br>5. In public places in Thailand<br>6. On the transport vehicle<br>7. I do not know / do not remember<br>8. I do not want to answer	
151.	Have you ever had anyone who <u>threaten to harm you or someone you care about</u>?	1. Yes<br>**2. No**<br>**3. I do not know / do not remember**<br>**4. I do not want to answer**	 →Q156<br>→Q156<br>→Q156

	For women who have ever had a partner, please mention this fact if required: Your partner is not included.		
152.	Would you say that such kind of event happened one time or a few times or many times?	1. Once<br>2. A few times<br>3. Many times<br>4. I do not know / do not remember<br>5. I do not want to answer	
153.	Who did it?	*Select all that apply*<br>1. A male member of the respondent's family<br>2. Female member of the respondent's family<br>3. Male employer<br>4. Female employer<br>5. Male colleague<br>6. Female colleague<br>7. Male stranger<br>8. Stranger woman<br>9. Migration / immigration Officer<br>10. Jobseeker or broker<br>11. Police officer<br>12. Soldier<br>13. Soldier who is not from the State Army<br>14. Other ___<br>15. I do not know / do not remember<br>16. I do not want to answer	
154.	Did this happen in the last 12 months (March 2020 until now) or before the past 12 months or both?	1. Only in the last 12 months (March 2020 until now)<br>2. Only before 12 months ago<br>3. Both in the last 12 months and within the last 12 months<br>4. I do not know / do not remember<br>5. I do not want to answer	
155.	Where did it happen?	*Select all that apply*<br>1. In my home country<br>2. While traveling to your home country and Thailand<br>3. At my workplace in Thailand<br>4. At my place of residence in Thailand<br>5. In public places in Thailand<br>6. On the transport vehicle<br>7. I do not know / do not remember<br>8. I do not want to answer	
156.	Has anyone ever <u>checked your whereabouts using mobile technology or social media</u>?<br>For women who have ever had a partner, please mention this fact if required: Your partner is not included.	1. Yes<br>**2. No**<br>**3. I do not know / do not remember**<br>**4. I do not want to answer**	 →Q161<br>→Q161<br>→Q161
157.	Would you say that such kind of event happened one time or a few times or many times?	1. Once<br>2. A few times<br>3. Many times<br>4. I do not know / do not remember<br>5. I do not want to answer	
158.	Who did it?	*Select all that apply*<br>1. A male member of the respondent's family<br>2. Female member of the respondent's family<br>3. Male employer<br>4. Female employer	

		5. Male colleague<br>6. Female colleague<br>7. Male stranger<br>8. Stranger woman<br>9. Migration / immigration Officer<br>10. Jobseeker or broker<br>11. Police officer<br>12. Soldier<br>13. Soldier who is not from the State Army<br>14. Other ___<br>15. I do not know / do not remember<br>16. I do not want to answer	
159.	Did this happen in the last 12 months (March 2020 until now) or before the past 12 months or both?	1. Only in the last 12 months (March 2020 until now)<br>2. Only before 12 months ago<br>3. Both in the last 12 months and within the last 12 months<br>4. I do not know / do not remember<br>5. I do not want to answer	
160.	Where did it happen?	*Select all that apply*<br>1. In my home country<br>2. While traveling to your home country and Thailand<br>3. At my workplace in Thailand<br>4. At my place of residence in Thailand<br>5. In public places in Thailand<br>6. On the transport vehicle<br>7. I do not know / do not remember<br>8. I do not want to answer	
161.	Has anyone ever <u>checked your SMS, messages from social media or phone calls without your permission</u>?<br>For women who have ever had a partner, please mention this fact if required: Your partner is not included.	1. Yes<br>**2. No**<br>**3. I do not know / do not remember**<br>**4. I do not want to answer**	 →Q166<br>→Q166<br>→Q166
162.	Would you say that such kind of event happened one time or a few times or many times?	1. Once<br>2. A few times<br>3. Many times<br>4. I do not know / do not remember<br>5. I do not want to answer	
163.	Who did it?	*Select all that apply*<br>1. A male member of the respondent's family<br>2. Female member of the respondent's family<br>3. Male employer<br>4. Female employer<br>5. Male colleague<br>6. Female colleague<br>7. Male stranger<br>8. Stranger woman<br>9. Migration / immigration Officer<br>10. Jobseeker or broker<br>11. Police officer<br>12. Soldier<br>13. Soldier who is not from the State Army<br>14. Other ___<br>15. I do not know / do not remember<br>16. I do not want to answer	

164.	Did this happen in the last 12 months (March 2020 until now) or before the past 12 months or both?	1. Only in the last 12 months (March 2020 until now)<br>2. Only before 12 months ago<br>3. Both in the last 12 months and within the last 12 months<br>4. I do not know / do not remember<br>5. I do not want to answer	
165.	Where did it happen?	*Select all that apply*<br>1. In my home country<br>2. While traveling to your home country and Thailand<br>3. At my workplace in Thailand<br>4. At my place of residence in Thailand<br>5. In public places in Thailand<br>6. On the transport vehicle<br>7. I do not know / do not remember<br>8. I do not want to answer	
166.	Have you ever been <u>beaten or physically abused by someone</u>?<br>For women who have ever had a partner, please mention this fact if required: Your partner is not included.	1. Yes<br>**2. No**<br>**3. I do not know / do not remember**<br>**4. I do not want to answer**	 →Q171<br>→Q171<br>→Q171
167.	Would you say that such kind of event happened one time or a few times or many times?	1. Once<br>2. A few times<br>3. Many times<br>4. I do not know / do not remember<br>5. I do not want to answer	
168.	Who did it?	*Select all that apply*<br>1. A male member of the respondent's family<br>2. Female member of the respondent's family<br>3. Male employer<br>4. Female employer<br>5. Male colleague<br>6. Female colleague<br>7. Male stranger<br>8. Stranger woman<br>9. Migration / immigration Officer<br>10. Jobseeker or broker<br>11. Police officer<br>12. Soldier<br>13. Soldier who is not from the State Army<br>14. Other ___<br>15. I do not know / do not remember<br>16. I do not want to answer	
169.	Did this happen in the last 12 months (March 2020 until now) or before the past 12 months or both?	1. Only in the last 12 months (March 2020 until now)<br>2. Only before 12 months ago<br>3. Both in the last 12 months and within the last 12 months<br>4. I do not know / do not remember<br>5. I do not want to answer	
170.	Where did it happen?	*Select all that apply*<br>1. In my home country<br>2. While traveling to your home country and Thailand<br>3. At my workplace in Thailand<br>4. At my place of residence in Thailand<br>5. In public places in Thailand	

		6. On the transport vehicle<br>7. I do not know / do not remember<br>8. I do not want to answer	
171.	Have you ever been experienced any of the followings done by anyone: <u>Using or threatening with a knife or other weapon</u>?<br>For women who have ever had a partner, please mention this fact if required: Your partner is not included.	1. Yes<br>**2. No**<br>**3. I do not know / do not remember**<br>**4. I do not want to answer**	 →Q176<br>→Q176<br>→Q176
172.	Would you say that such kind of event happened one time or a few times or many times?	1. Once<br>2. A few times<br>3. Many times<br>4. I do not know / do not remember<br>5. I do not want to answer	
173.	Who did it?	*Select all that apply*<br>1. A male member of the respondent's family<br>2. Female member of the respondent's family<br>3. Male employer<br>4. Female employer<br>5. Male colleague<br>6. Female colleague<br>7. Male stranger<br>8. Stranger woman<br>9. Migration / immigration Officer<br>10. Jobseeker or broker<br>11. Police officer<br>12. Soldier<br>13. Soldier who is not from the State Army<br>14. Other ___<br>15. I do not know / do not remember<br>16. I do not want to answer	
174.	Did this happen in the last 12 months (March 2020 until now) or before the past 12 months or both?	1. Only in the last 12 months (March 2020 until now)<br>2. Only before 12 months ago<br>3. Both in the last 12 months and within the last 12 months<br>4. I do not know / do not remember<br>5. I do not want to answer	
175.	Where did it happen?	*Select all that apply*<br>1. In my home country<br>2. While traveling to your home country and Thailand<br>3. At my workplace in Thailand<br>4. At my place of residence in Thailand<br>5. In public places in Thailand<br>6. On the transport vehicle<br>7. I do not know / do not remember<br>8. I do not want to answer	
176.	Have you ever experienced any of the followings from anyone: <u>Tracking you; Sending sexually explicit messages you do not want (via SMS, email, or social media); Sexual alertness or obsession that you do not want</u>?<br>For women who have ever had a partner, please mention this fact if required: Your partner is not included.	1. Yes<br>**2. No**<br>**3. I do not know / do not remember**<br>**4. I do not want to answer**	 →Q181<br>→Q181<br>→Q181

177.	Would you say that such kind of event happened one time or a few times or many times?	1. Once<br>2. A few times<br>3. Many times<br>4. I do not know / do not remember<br>5. I do not want to answer	
178.	Who did it?	*Select all that apply*<br>1. A male member of the respondent's family<br>2. Female member of the respondent's family<br>3. Male employer<br>4. Female employer<br>5. Male colleague<br>6. Female colleague<br>7. Male stranger<br>8. Stranger woman<br>9. Migration / immigration Officer<br>10. Jobseeker or broker<br>11. Police officer<br>12. Soldier<br>13. Soldier who is not from the State Army<br>14. Other ___<br>15. I do not know / do not remember<br>16. I do not want to answer	
179.	Did this happen in the last 12 months (March 2020 until now) or before the past 12 months or both?	1. Only in the last 12 months (March 2020 until now)<br>2. Only before 12 months ago<br>3. Both in the last 12 months and within the last 12 months<br>4. I do not know / do not remember<br>5. I do not want to answer	
180.	Where did it happen?	*Select all that apply*<br>1. In my home country<br>2. While traveling to your home country and Thailand<br>3. At my workplace in Thailand<br>4. At my place of residence in Thailand<br>5. In public places in Thailand<br>6. On the transport vehicle<br>7. I do not know / do not remember<br>8. I do not want to answer	
181.	Has anyone ever <u>sexually assaulted you or made you touch their body parts</u>? It involves touching the breast or other parts of the body.<br>For women who have ever had a partner, please mention this fact if required: Your partner is not included.	1. Yes<br>**2. No**<br>**3. I do not know / do not remember**<br>**4. I do not want to answer**	 →Q186<br>→Q186<br>→Q186
182.	Would you say that such kind of event happened one time or a few times or many times?	1. Once<br>2. A few times<br>3. Many times<br>4. I do not know / do not remember<br>5. I do not want to answer	
183.	Who did it?	*Select all that apply*<br>1. A male member of the respondent's family<br>2. Female member of the respondent's family<br>3. Male employer<br>4. Female employer<br>5. Male colleague	

		6. Female colleague<br>7. Male stranger<br>8. Stranger woman<br>9. Migration / immigration Officer<br>10. Jobseeker or broker<br>11. Police officer<br>12. Soldier<br>13. Soldier who is not from the State Army<br>14. Other ___<br>15. I do not know / do not remember<br>16. I do not want to answer	
184.	Did this happen in the last 12 months (March 2020 until now) or before the past 12 months or both?	1. Only in the last 12 months (March 2020 until now)<br>2. Only before 12 months ago<br>3. Both in the last 12 months and within the last 12 months<br>4. I do not know / do not remember<br>5. I do not want to answer	
185.	Where did it happen?	*Select all that apply*<br>1. In my home country<br>2. While traveling to your home country and Thailand<br>3. At my workplace in Thailand<br>4. At my place of residence in Thailand<br>5. In public places in Thailand<br>6. On the transport vehicle<br>7. I do not know / do not remember<br>8. I do not want to answer	
186.	Have you ever been forced to do any sexual acts from anyone (except from your partner): e.g. Threatening you; Controlling you or getting you to the point where you can't deny it?<br>Be careful to include people you know, including strangers.	1. Yes<br>**2. No**<br>**3. I do not know / do not remember**<br>**4. I do not want to answer**	 →Q191<br>→Q191<br>→Q191
187.	Would you say that such kind of event happened one time or a few times or many times?	1. Once<br>2. A few times<br>3. Many times<br>4. I do not know / do not remember<br>5. I do not want to answer	
188.	Who did it?	*Select all that apply*<br>1. A male member of the respondent's family<br>2. Female member of the respondent's family<br>3. Male employer<br>4. Female employer<br>5. Male colleague<br>6. Female colleague<br>7. Male stranger<br>8. Stranger woman<br>9. Migration / immigration Officer<br>10. Jobseeker or broker<br>11. Police officer<br>12. Soldier<br>13. Soldier who is not from the State Army<br>14. Other ___<br>15. I do not know / do not remember<br>16. I do not want to answer	

189.	Did this happen in the last 12 months (March 2020 until now) or before the past 12 months or both?	1. Only in the last 12 months (March 2020 until now)<br>2. Only before 12 months ago<br>3. Both in the last 12 months and within the last 12 months<br>4. I do not know / do not remember<br>5. I do not want to answer	
190.	Where did it happen?	*Select all that apply*<br>1. In my home country<br>2. While traveling to your home country and Thailand<br>3. At my workplace in Thailand<br>4. At my place of residence in Thailand<br>5. In public places in Thailand<br>6. On the transport vehicle<br>7. I do not know / do not remember<br>8. I do not want to answer	
191.	Have you ever been asked or pressured into engaging in sexual misconduct or accepting sexual favors in order to get a job or to continue working or to be promoted?	1. Yes<br>2. No<br>3. I do not know / do not remember<br>4. I do not want to answer	
192.	Have you ever experienced a police officer/ soldier/ illegal military/ insurgent fighters threatening or intimidating someone else in your home country?	1. I have ever seen<br>2. I have never seen it<br>3. I do not know / do not remember<br>4. I do not want to answer	
Assessment: If you have never witnessed or witnessed violence in home country, please skip to the S5			
193.	Is this one of the reasons for your migration to Thailand? (Your experience in your home country that a police officer/ soldier/ illegal military/ insurgent fighters threatening or intimidating you or someone else)	1. Absolutely not<br>2. Somewhat yes<br>3. Absolutely yes<br>4. Not applicable to me / Never experienced such violence. I have never seen it<br>5. I do not know / do not remember<br>6. I do not want to answer	

#	Section 5: Impact		Skip
**Assessment: If you have never experienced any violence, please skip to the S6.**			
194.	How do you feel about these difficult experiences that you have told me?	*Select all that apply*<br>1. It has no effect on how I feel<br>2. I feel ashamed or embarrassed<br>3. Feeling scared or insecure or worried about what might happen next<br>4. Concerned about the safety of children<br>5. Sad, feeling hopeless or depressed<br>6. Feelings of anger<br>7. Other _____<br>8. I do not know/do not remember<br>9. I do not want to answer	

195.	If applicable, in what way did these difficult experiences that you have told me affect adverse effects on your job or your lucrative business?	*Select all that apply*<br>1. Did not affect work<br>2. I was distracted from work, but not had to take a break from work<br>3. I could not focus on work<br>4. Could not work. I had to take a break from work<br>5. Loss of confidence in my abilities<br>6. I lost my job<br>7. I had to change departments / sections in the workplace<br>8. I had to change job / employer<br>9. Other ______<br>10. I do not know / do not remember<br>11. I do not want to answer	
196.	Have you <u>ever had</u> a physical injury or illness as a result of these events? Please think back to the actions we mentioned earlier.	1. Yes<br>**2. No**<br>**3. I do not know / do not remember**<br>**4. I do not want to answer**	 →S6<br>→S6<br>→S6
197.	Have you ever received medical care for such an injury/ illness (s)? Sometimes or always or never?	1. I have received; Sometimes.<br>2. I have received; Always.<br>3. No; Never.<br>4. I do not know / do not remember<br>5. I do not want to answer	

#	Section 6: Coping and knowledge		SKIP
Some of these questions are very difficult to answer. Thank you for answering. Your answers are very important. In this section I would like to ask you some questions about how to keep yourself safe.			
198.	Have you ever told someone or asked for help when you were experiencing these experiences?	1. Yes<br>**2. No**<br>**3. I do not know / do not remember**<br>**4. I do not want to answer**	 →Q201<br>→Q201<br>→Q201
199.	To whom did you tell / ask for help?	*Select all that apply*<br>1. Family<br>2. Friends<br>3. Employer<br>4. Colleagues<br>5. Police<br>6. Healthcare workers<br>7. Civil Society / Support Services<br>8. Religious leader<br>9. Social Media<br>10. Other __________<br>11. I do not know / do not remember<br>12. I do not want to answer	
200.	How did they help you?	*Select all that apply*<br>1. Gathered others to help<br>2. Intervened<br>3. I was taken for medical treatment<br>4. Informed the police<br>5. Informed the employer / manager<br>6. Took me to a civil society or support service or made a hotline call<br>7. Provided a safe place to live<br>8. Gave me money/ Provided clothing and other items<br>9. Asked me how I would like to get help	

		10. Talk to the perpetrators who committed to me<br>11. Did not help me<br>12. Other: ______<br>13. I do not know / do not remember<br>14. I do not want to answer	
201.	To whom would you tell / ask for help if you will have similar experiences in the future?	*Select all that apply*<br>1. My family<br>2. My friends<br>3. My employer<br>4. My colleagues<br>5. Police<br>6. Healthcare workers<br>7. Civil Society / Support Services<br>8. Religious leader<br>9. Social Media<br>10. Other __________<br>11. I do not know / do not remember<br>12. I do not want to answer	
202.	Do you know where to go for help if a female migrant worker is beaten or abused?	1. Yes<br>**2. No**<br>**3. I do not want to answer**	 →S7<br>→S7
203.	If this happens in girls/women, where in the community can they go for help?	*Do not read the answers.*<br>*Please select all that apply.*<br>1. Family<br>2. Friends<br>3. Thai police<br>4. Healthcare center/clinic<br>5. Where a civil society or support service is provided<br>6. Hotline<br>7. Employer<br>8. Colleagues / other migrant workers<br>9. Religious leader<br>10. Other: _____<br>11. I do not know / do not remember<br>12. I do not want to answer	

#	Section 7: Socio-Economic Situation		skip
If you do not mind, I would like to ask a few questions about your family situation. These are the last questions.			
204.	How often do families in your home country suffer from food shortages because they have no money?	1. Every week<br>2. Every month, but not every week<br>3. It is happening. Not every month<br>4. Never<br>5. I do not know / do not remember<br>6. 6. I do not want to answer	
205.	If Money is urgently needed; 3,000 baht is needed for emergencies. For example – funeral. If so, how easy is it for you to find that money?	1. Very difficult<br>2. Somewhat difficult<br>3. Easy on average<br>4. Very easy<br>5. I do not know / do not remember<br>6. I do not want to answer	
206.	Do family members in your household own land?	1. Yes<br>**2. No**<br>**3. I do not know / do not remember**<br>4. **I do not want to answer**	

207.	We are now done with our interview. Do you have any comments? Or do you want to add something?	1. [Write comments from respondents]	
208.	I have asked you difficult questions that you may be difficult to answer. How do you feel after saying that?	1. Good / better<br>2. Bad / worse<br>3. Same / nothing else changed	

FINISH OPTION 1: IF RESPONDENT HAS DISCLOSED EXPERIENCING VIOLENCE
Thank you for taking the time to share your story with me today. I realize that some of these questions may have been difficult for you to answer, but it is only by hearing from women themselves that we can really understand about their health and experiences of violence.

From what you have told us, I can tell that you have had some very difficult times in your life. No one has the right to treat someone else in that way. However, from what you have told me I can see also that you are strong and have survived through some difficult circumstances. The information you have shared with me will be used to help improve the experiences of migrant women workers such as yourself.

[PROVIDE PARTICIPANT WITH SERVICE CARD] *Here is a list of organizations that provide support, legal advice and counselling services to women in Thailand. Please do contact them if you would like to talk over your situation with anyone. Their services are free, and they will keep anything that you say confidential. You can go whenever you feel ready to, either soon or later on.*

FINISH OPTION 2: IF RESPONDENT HAS NOT DISCLOSED EXPERIENCING VIOLENCE
Thank you for taking the time to share your story with me today. I realize that some of these questions may have been difficult for you to answer, but it is only by hearing from women themselves that we can really understand about women's health and experiences in life. The information you have shared with me will be used to help improve the experiences of migrant women workers such as yourself.

[PROVIDE PARTICIPANT WITH SERVICE CARD] In case you ever hear of another woman who needs help, here is a list of organizations that provide support, legal advice and counselling services to women in Thailand. Please do contact them if you or any of your friends or relatives need help. Their services are free, and they will keep anything that anyone says to them confidential.
